# The design and development of covalent protein-protein interaction inhibitors for cancer treatment

**DOI:** 10.1186/s13045-020-00850-0

**Published:** 2020-03-30

**Authors:** Sha-Sha Cheng, Guan-Jun Yang, Wanhe Wang, Chung-Hang Leung, Dik-Lung Ma

**Affiliations:** 1 Institute of Chinese Medical Sciences, State Key Laboratory of Quality Research in Chinese Medicine, University of Macau, Macao, SAR China; 2 Department of Chemistry, Hong Kong Baptist University, Kowloon, 999077 Hong Kong, China; 3 Institute of Medical Research, Northwestern Polytechnical University, Xi’an, 710072 China

**Keywords:** Protein-protein interaction, Covalent inhibitors, Cancer therapy

## Abstract

Protein-protein interactions (PPIs) are central to a variety of biological processes, and their dysfunction is implicated in the pathogenesis of a range of human diseases, including cancer. Hence, the inhibition of PPIs has attracted significant attention in drug discovery. Covalent inhibitors have been reported to achieve high efficiency through forming covalent bonds with cysteine or other nucleophilic residues in the target protein. Evidence suggests that there is a reduced risk for the development of drug resistance against covalent drugs, which is a major challenge in areas such as oncology and infectious diseases. Recent improvements in structural biology and chemical reactivity have enabled the design and development of potent and selective covalent PPI inhibitors. In this review, we will highlight the design and development of therapeutic agents targeting PPIs for cancer therapy.

## Background

The protein-protein interaction (PPI) is defined as a physical link between a protein and its partner(s) [1–3]. These connections may display a range of heterogeneities and complexities in macromolecular structures, forming protein dimers, multicomponent complexes, or long chains [4]. The interaction between protein subunits can be transient or permanent, identical or heterogeneous, and specific or nonspecific [3, 5, 6]. There are nearly 650,000 PPIs in humans, and this number continues to increase as more interaction networks become discovered [3, 7]. The function of proteins plays an essential role in the context of PPI networks [5]. For example, the PPI system connects different enzymes with their protein substrates and regulates the activity of proteins [5]. Twenty percent of proteins exist in network hubs and interact with at least 24 partners [8]. Proteins occupy almost half of the dry mass of a cell, and the disruption of PPIs often causes diseases, including cancer [9, 10]. Hence, research on PPI plays a central role in progressing our understanding of molecular biology and human diseases, as well as for developing new therapeutic agents in drug discovery [6, 11, 12].

**Fig. 1:**
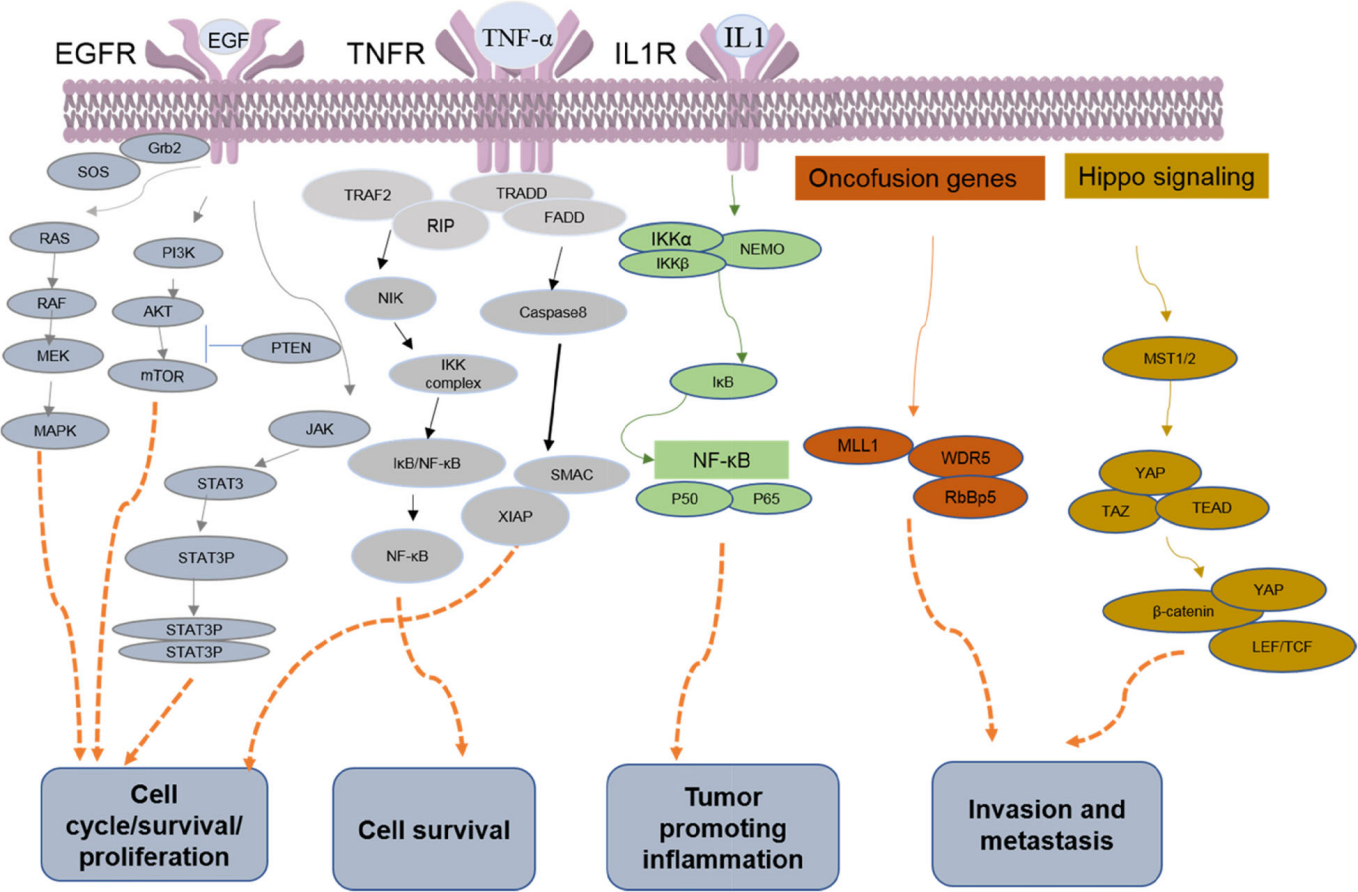
Oncogenic PPI networks that are associated with the hallmarks of tumorigenesis. It should be noted that some PPI networks regulate global mechanisms of cell growth and their relationship to cancer remains to be verified

The abnormal regulation of PPIs contributes to the majority of cancers. PPIs are involved in all phases of oncogenesis, from cell proliferation, cell survival, and inflammation to invasion and metastasis (Fig. 1) [13, 14]. Understanding the molecular mechanisms of PPIs is therefore crucial for developing accurate methods for the prevention, diagnosis, and treatment of cancers. The contact interface between two proteins is the structural foundation of their interaction. Understanding the contact region between proteins will help to elucidate their functions in interaction networks. It should be noted that similar or overlapping interfaces can be promiscuous and be employed many times in hub proteins [15]. The cancer-related proteins are abnormally expressed (overexpressed, low expressed, or mutant) in cancer cells compared to normal cells. For example, S100A13 overexpression contributed to tumor metastasis and poor survival in patients with early-stage non-small cell lung cancer [16]. Low TMEFF2 expression was associated with larger tumor size and advanced stage and poor differentiation in pancreatic cancer cells [17]. It was reported that more than 50% of cancer patients have p53 mutations, which may cause cancer therapy resistance, and the underlying mechanism is poorly understood [18]. Cancer-associated protein-protein interaction network which is involved in cancer development tend to interact with each other to form a cancer-specific interaction network, and it is important for acquisition and maintaining characteristics of cancer essential for cell transformation [19, 20]. Deeper investigations of protein-protein interfaces relevant to human oncogenesis and cancer-associated protein-protein interaction networks have shown that cancer-related proteins are smaller, more planar, more charged, and less hydrophobic binding sites than non-cancer-related proteins and they tend to show lower affinity and higher specificity for cancer-associated PPI networks. Moreover, cancer-related proteins often interface with their binding partners using distinct surfaces, corresponding typically to multi-interface hub [21]. Therefore, targeting PPIs can be a viable approach for cancer treatment since the aberrant activity of these networks often directly leads to tumor progression.

**Fig. 2:**
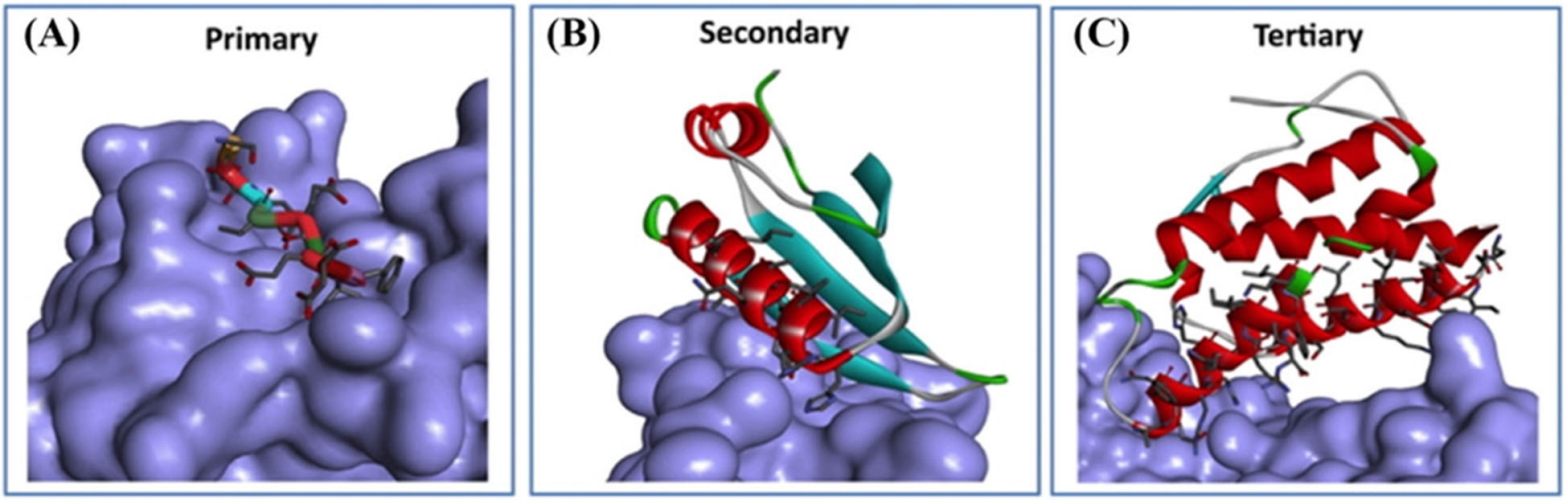
The crystal structure of three different classes of PPIs. **a** Linear sequences comprise primary peptide epitopes (e.g., LM of DNA polymerase III bound to the binding pocket of the SC (PDB: 3D1F)). **b** The secondary structure of epitopes binds as a single unit, e.g., an α-helix (NusB-NusE PPI (PDB: 3R2C)). **c** In tertiary structural epitopes, the binding interface is not continuous and both sides of PPI interface are needed (e.g., IL-2/IL-2Ra PPI (PDB 1Z92))

Compared with targeting enzymes or receptors, however, the development of molecules targeting PPIs has been challenging [22–24]. PPIs have relatively large and amorphous interfaces, rather than small and well-defined crevices. Recent efforts in developing clinical PPI inhibitors have focused on targeting “hotspots” that typically span 250–900 Å^2^ of the PPI interface [25]. Generally, there are three different classes of PPI: short continuous peptide epitopes, secondary structural epitopes, and tertiary structural epitopes. Short continuous peptide epitopes consist of continuous linear sequences of about 6–9 amino acids (Fig. 2a) [26]. Secondary structural epitopes can bind as single unit, for example, a single face of an α-helix binding to a hydrophobic groove of complementary residues (Fig. 2b) [27, 28]. In the tertiary structure of epitopes, the binding interface is not continuous and requires multiple sites to form the PPI interface [24]. Compared with primary and secondary structure of epitopes, the interfaces of tertiary epitopes are more dynamic and prevalent (Fig. 2c) [29]. Targeting the tertiary structural epitopes of PPIs with chemical agents is challenging, but may also represent a vast area of opportunity as well as they tend to be much more dynamic than the primary and secondary class epitopes. To date, many PPI modulators have been developed and some of these have even successfully entered the clinic [30–33]. PPI inhibitors can be classified into small molecules, antibodies, peptides, or recombinant proteins. As PPI interfaces are generally wide, therapeutic antibodies have attracted attention as candidates for PPI inhibition [34, 35]. On the other hand, small molecules are much cheaper and easier to produce compared to protein- or peptide-based drugs and may tolerate a wider range of administration modes (e.g., oral), which makes them favorites of pharmaceutical chemists [34]. But there are three obstacles for targeting PPIs with small molecules. First, the contact interfaces of PPIs typically contain an array of polar and non-polar interactions spread over a large area (1500–3000 Å^2^) [36]. Unless the interaction hotspot can be identified, a small molecule with a reduced footprint has difficulty achieving tight binding owing to insufficient interactions. Secondly, protein-protein interfaces are usually flat. Without concavity, a small molecule is limited to only contact one side of the binding site, which is comparatively less effective compared with inserting into a deep groove. Thirdly, targeting PPIs with covalent inhibitors is not possible if an appropriate nucleophilic residue is not available in the protein-binding interface, especially free cysteine, lysine, or methionine residues [32, 33]. Therefore, targeting alternative nucleophilic sites, including tyrosine, threonine, serine, and histidine, should be investigated to offer new avenues for covalent inhibitor development. Aided by recent advancements in structural biology and chemistry, covalent inhibitors have emerged as a potentially effective approach towards PPI inhibition [37, 38]. Studies have indicated that covalent drugs are less likely to promote resistance [39], which could be important for diseases such as cancer and infectious diseases where drug resistance is a major challenge. Covalent inhibitors are generally comprised of a specificity group to recognize the target protein and a functional “warhead” that forms a covalent linkage with the protein. Compared with noncovalent inhibitors, covalent PPI inhibitors can have the advantages of sustained inhibition and longer residence times, as covalently bound PPI targets are persistently inhibited until protein degradation and regeneration [40]. Additionally, covalent inhibitors can exhibit high selectivity for proteins with non-conserved residues between variant forms or protein family members [41]. This can be a significant advantage in cancer treatment, because point mutations of target proteins often generate resistance to drug treatment [42, 43]. Thus, covalent inhibitors targeting PPIs offer the potential ability for expanding the therapeutic range [44]. However, the strong binding of covalent inhibitors can also produce drawbacks. In the 1970s, researchers reported that covalent drugs produce hepatotoxicity resulting from off-target effects via unselectively modifying non-target proteins with their highly reactive groups [37, 45]. Meanwhile, whether inhibitors are covalent or non-covalent, side effects arising from idiosyncratic risks are always an issue that has to be considered in drug research and development [46, 47]. Given the potential of covalent drugs, the advantages and disadvantages of covalent PPI inhibitors should be carefully judged and weighed by practitioners in drug discovery.

### Strategies to design and develop covalent PPI inhibitors

#### Different strategies to design covalent PPI inhibitors

Different strategies are needed to design covalent PPI inhibitors compared to non-covalent inhibitors [48–50]. Covalent inhibitors traditionally target cysteine; however, their natural abundance is low and these residues have unique reactivity in proteome. Therefore, new strategies are imperative to design and develop to target interaction sites that lack reactive cysteine residues. The following sections discuss methods for the development of covalent PPI inhibitors, including structure-guided design, activity-based profiling, and covalent docking.

#### Structure-guided design of covalent inhibitors

Advancements in structural biology have aided the structure-guided design of non-covalent and covalent inhibitors alike [51]. Covalent PPI inhibitors typically react with a side chain of an amino acid that is located within the binding site to form a covalent bond, which are usually (but not always) irreversible. In traditional drug discovery, the linkage between a target and its corresponding disease drives the development of therapeutic molecules against the target. For the structure-based design of covalent inhibitors, an alternative prospective strategy has been considered that first identifies potential proteins that could be targeted by covalent inhibitors, which are then consolidated into target subsets. Recently, a new technology to identify covalent fragments has emerged, which uses reactive covalent ligands to identify covalent targets in phenotypic or target-based screens [52].

Reactive cysteine residues on proteins have high nucleophilicity, which enables covalent binding with electrophilic small molecules [53, 54]. Therefore, reactive cysteine residues at the PPI site are considered as a common characteristic for the design of covalent PPI inhibitors. Computational tools can detect covalent binding site through several methods: (1) binding site prediction, (2) hydrogen bond frequency analysis, and (3) covalent binding site detection [55]. Meanwhile, comprehensive databases including targetable cysteine and known covalent inhibitors can be manually curated [54–57]. However, it should be noted that covalent inhibitors may modify many available residues in a target, and some proteins may be more susceptible to this than others. Therefore, it is vital to accurately pinpoint a residue’s location in the target as well as in similar binding sites as this could influence the agents’ selectivity.

Interaction binding pockets in protein structures can also be identified through computational algorithms [58]. Using a structure identification approach, various potential interaction pockets can be ranked in terms of the surrounding residues. There are some successful cases demonstrating that analyzing the residues around the PPI binding site can provide useful information for covalent inhibitor development. For instance, the peptides second mitochondria-derived activator of caspase (SMAC) and N-terminal tetrapeptide of the sequence Ala-Val-Pro-Phe (AVPF), discovered through structure-based approaches, have high stability, affinity, and cell permeability, and ultimately increase the drug-likeness of the original tetrapeptide. Some potential drug-like peptides of SMAC AVPF have been applied in various treatment areas and some have even entered clinical trials [59]. This successful case of structure-based design provides a promising prospective for designing covalent inhibitors against PPIs. Studies on structural and cellular reveal that when mitochondrial release into the cytosol, the activation of SMACs exposes the N-terminal tetrapeptide of the sequence AVPF. The tetrapeptide mediates its interactions with various members of the IAP family, including the X-linked inhibitor of apoptosis protein (XIAP). In addition, Lys-covalent inhibitors have been designed with a benzamide-sulfonyl fluoride warhead to target the BIR3 domain of XIAP [60, 61].

In another study, the structure-based biophysical and biochemical approaches were used to develop different kinds of warheads which can easily form covalent adducts with alternative amino acids such as histidine, threonine, serine, and tyrosine [62]. It was found that aryl-fluoro sulfate electrophiles can form covalent bonds with Lys, Tyr, and His residues in PPIs [61]. With the tremendous progress and resurgence for covalent drugs, these successful cases present valuable and novel ideas for covalent PPI inhibitors and suggest the possibility for extending the target cysteinome range to other residues.

#### Activity-based protein profiling of covalent inhibitors

**Fig. 3:**
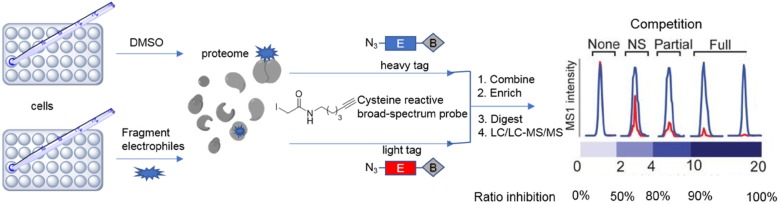
The general workflow of the isoTOP-ABPP platform. The cell proteome is labeled with a cysteine-reactive probe, and then samples are conjugated to TEV protease-cleavable biotin tags (heavy (blue) and light (red)). Competition ratios are calculated following protein digestion and multidimensional liquid chromatography-tandem mass spectrometry

Activity-based protein profiling (ABPP) is a type of chemical proteome method that aims to evaluate the on-target and off-target activities of a covalent drug. In ABPP, activity-based probes (ABPs) are used to visualize the target protein in a mechanism-based fashion [63]. By targeting only the activated residue on the protein, only the catalytically active form of enzyme will be covalently labeled by the probe. In addition, ABPP methods are also applied to study protein activity at different stages of diseases, recognize and characterize various function of proteins including unknown ones, identify covalent linkages between protein targets and natural products, and identify potential inhibitors of protein [64]. In order to develop a site-specific technique for quantification of reactivity, researchers have developed a new method termed isotopic tandem orthogonal proteolysis (isoTOP)-ABPP [65]. A chemical-proteomic employing of isoTOP-ABPP was reported to analyze the cysteine activity of complicated proteomes (Fig. 3) [64]. IsoTOP-ABPP methods provide two opportunities for developing covalent inhibitors of PPI for drug discovery. Firstly, the method enables the identification of cysteines that can be targeted by covalent modification to regulate protein activity. Secondly, the technique can identify related targets in the cell in order to evaluate the promiscuity of small molecules, electrophilic groups, or natural products [65]. This assay can also be adapted to a competition mode, by challenging ligandable sites with both covalent drugs and reactive cysteines in order to aid rapid target discovery as well as discover new druggable hotspots that can be targeted by covalent drugs. In our view, the development of PPI covalent inhibitors could be accelerated by combining the platforms of isoTOP-ABPP with cysteine-reactive ligand libraries [66]. The approach can lead to the identification of covalent inhibitors targeting novel and complicated PPI binding sites, as well as for studying for cysteine reactivity in biological systems. Furthermore, other amino acids with reactive functional groups can also be studied using the chemical-proteomic platform of isoTOP-ABPP in order to identify new druggable hotspots in PPI. For example, dichlorotriazines have a tendency to bind with lysine [67], so they can be exploited as reactivity-based probes in ABPP platforms to detect and identify covalent inhibitors of PPIs [68]. Recently, dichlorotriazine-based covalent ligands were studied for their effects on triple-negative breast cancer cell survival [69]. The compound KEA1-97, identified using a ABPP-based chemoproteomic platform, targeted the lysine-72 site of thioredoxin to disrupt its PPI with caspase-3, leading to the activation of apoptosis [70]. Thus, expanding the application of ABPP with covalent ligand libraries targeting different amino acids (lysine, cysteine, methionine) could open new avenues for identifying covalent PPI drugs in the future. Moreover, these strategies also allow the identification of druggable hotspots and PPI targets for various diseases.

### Covalent docking

Computational tools can aid in the understanding of the complex interactions in biological systems [71]. The feasibility of hotspots as well as therapeutic targets can be evaluated through different computational strategies, for instance, molecular dynamics (MD), homology modeling, quantitative structure-activity relationships (QSAR), and molecular docking [72, 73]. However, most methods were initially developed for noncovalent inhibitors. Some of these can be applied for covalent drug design; however, configurations may have to be changed to allow for the formation of covalent bonds between the substrate and ligands [71].

**Fig 4:**
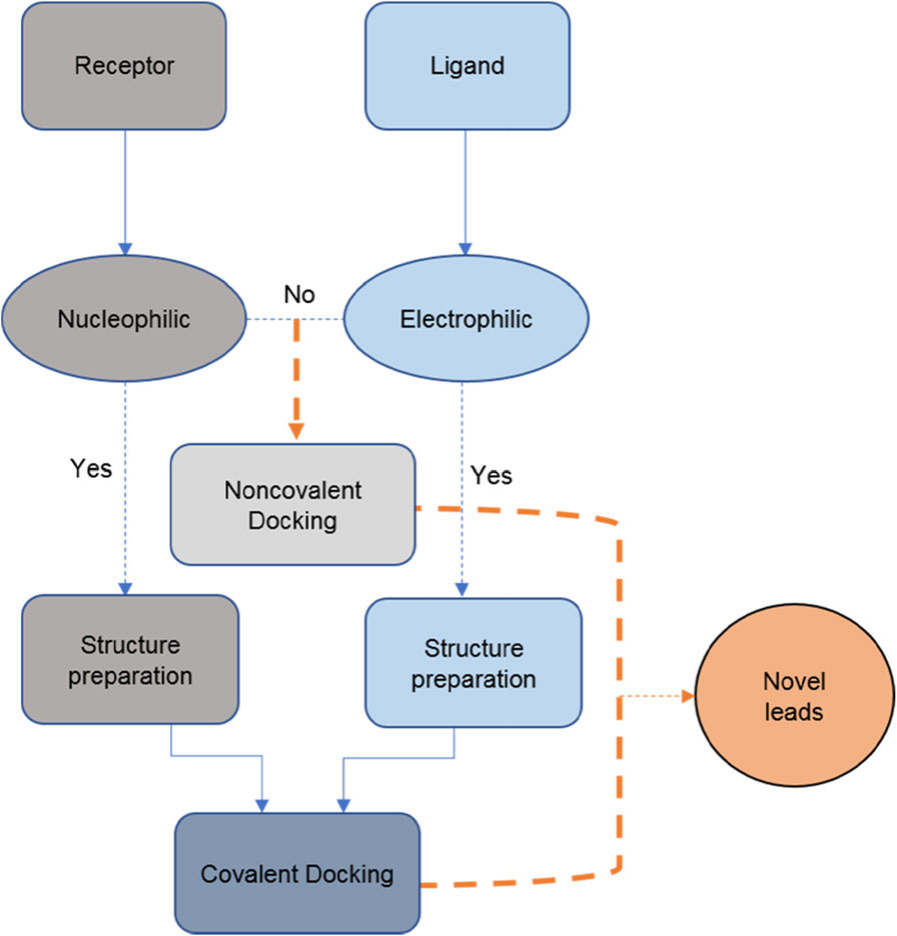
General scheme describing the workflow of covalent docking in drug discovery

Various kinds of algorithms have been developed to model covalent bonds between inhibitors and protein targets. In molecular docking system, the relative binding affinity of inhibitors and their binding mode can be predicted using the following two-step methodology. First, the conformational space is sampled for both the covalent ligand (usually flexible) and the protein (sometimes flexible). Second, the potential binding modes between the covalent ligands and the complexes are analyzed. Different covalent docking programs are available to identify covalent inhibitors of PPIs, such as MOE, CovDock, AutoDock4, FITTED, GOLD, and ICM-Pro [73]. Different protocols have been generated to covalently dock small molecules to biomolecular targets. However, the majority of covalent algorithms are limited to accurately calculating the binding energy between a nucleophilic protein and an electrophilic small molecule (Fig. 4) [74]. The “link atom” approach in GOLD is one of the most common routines for identifying possible linkages between a covalent ligand and the target protein in order to mimic the progress of covalent binding [73]. Autodock4 docks covalent inhibitors using either the “two-point attractor method” or “flexible side chain-based modification.” The former identifies and mimics the alkylating molecule as a free ligand in order to consider covalent bond formation between the ligand and an alkylated residue. The latter involves sampling the space around a single flexible side chain in order to assess the interaction between the protein attachment point and the covalent ligand [75]. Covalent docking also has some disadvantages, such as poor scoring functions, and limitations in torsional entropy, speed, and accuracy. The flexibility of side chains also needs to be accurately modeled in order to reflect the reality of the PPI binding site [76, 77]. Moreover, covalent docking requires substantial preparatory work that is not easily amenable to automation. The flexibility of side chains is another important factor to consider for covalent docking [76].

In the literature, covalent docking has been used to identify covalent PPI inhibitors in drug discovery. For example, the natural product andrographolide contains a reaction α,β-unsaturated lactone group, which targeted the nucleophilic Cys62 on the p50 subunit of NF-κB [78]. After performing computer docking, site-directed mutagenesis, and mass spectrometry, the researchers determined that the initial non-covalent interaction between inhibitors and residues on binding site are important for andrographolide covalent incorporation. This highlights the important fact that apart from the electrophilicity of the ligand, the redox environment and non-covalent interactions within the binding site should also be taken into account when designing covalent PPI inhibitors.

Some of the techniques for discovering covalent PPI inhibitors described above also have their own limitations. For example, the isoTOP-ABPP method is limited by the range of identifiable cellular cysteines and cannot evaluate the selectivity of compound in the total cellular cysteinome. Cell lysates are usually treated under non-reducing conditions, and so, some reactive cysteines may oxidize or aggregate. These shortcomings could lead to poor selectivity of compounds and aggregation or oxidation of reactive cysteines. These limitations can potentially be overcome by developing new cysteine-reactive electrophiles and other cell-based profiling methods [64]. Covalent docking also has some disadvantages, such as poor scoring functions, and limitations in speed and accuracy.

### Screening methods for developing covalent PPI inhibitors

In the past few years, a large number of covalent screening technologies have been developed and implemented for drug discovery. In the context of covalent PPI inhibitors, a variety of covalent high-throughput screening (HTS) methods can be used to complement conventional PPI screening assays in order to accelerate the identification of covalent PPI inhibitors.

The covalent binding of low molecular weight fragments can be identified by mass spectrometry or X-ray crystallography. X-ray crystallography allows for the direct verification of hits identified by the primary screening, since the crystal structure allows direct determination of the binding position of the fragment. Together with mutational analysis, NMR and X-ray crystallographic methods can be employed to identify hot spots in PPIs. After confirming the covalent binding mode of the fragment and identifying the hotspots targeted by the fragment, the affinity and selectivity of the compound can be further improved using traditional medicinal chemistry principles. Generally, covalent fragments have a relatively simple structure, and the reactivity of the fragment is the main factor driving binding activity of the PPI target. However, an excessively high binding reactivity could also increase the risk of off-target toxicity. During optimization, a decrease in the reactivity of the covalent warhead may be required to improve selectivity for a particular residue over others [40, 79].

Besides screening fragment-sized covalent ligands, it is also possible to perform HTS on a larger number of larger molecule weight and more drug-like covalent inhibitors. Having a larger library enables testing of a wide range reactivity of warheads, including those that may form reversible linkages to the target. In one example, covalent PPI inhibitors were identified against RAS G12C [80]. After identifying the hit of covalent inhibitors through fragment-based screening, a structure-based drug design strategy was used to improve their selectivity and efficiency. However, a limitation of HTS for covalent screening is that a large number of covalent inhibitors are needed in the library. Moreover, enough protein construct and appropriate identification methods are also essential for screening covalent binding between protein targets and PPI inhibitors. Covalent inhibitors targeting PPIs can be also screened through phenotypic or “black-box” methods. Many classic covalent inhibitors have been identified by this method. In order to evaluate the phenotypic changes at the cellular level, an appropriate cellular assay is required. There are two main advantages of the phenotypic screening method for identifying covalent PPI inhibitors. Firstly, this technique identifies cell-active compounds directly. Secondly, the biological target does not have to be known before screening [81]. However, disadvantages are that a sufficiently large covalent library is needed and that the target will have to be eventually identified in order to optimize the selectivity of the drug.

**Fig. 5:**
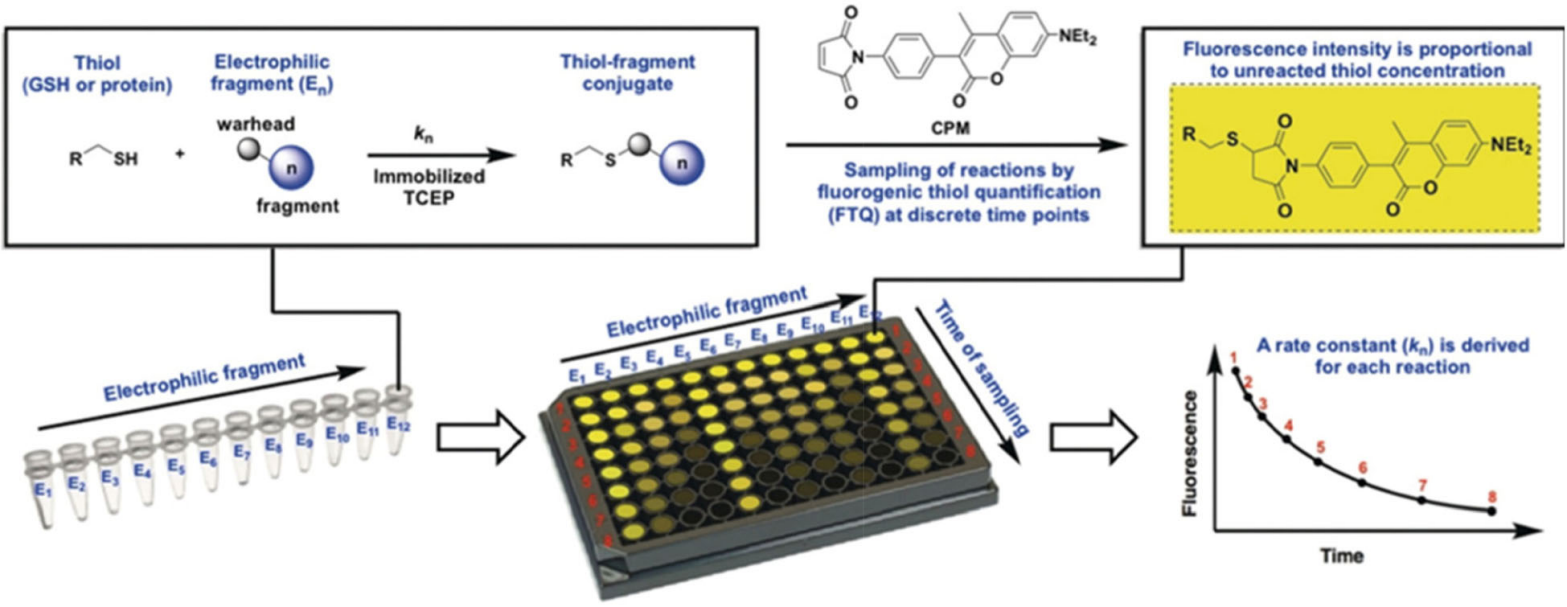
The workflow of the qIT assay. The thiol is challenged with electrophiles under pseudo-first-order conditions in the presence of TCEP. The reaction progress is monitored by analyzing residual thiol concentration using the fluorogenic probe CPM

Recently, the quantitative irreversible tethering (qIT) method has been reported for screening PPI covalent inhibitors. This approach is based on kinetic selectivity maximization in order to identify small molecule covalent inhibitors with cysteine reactivity. In this assay, thiol consumption is analyzed in a high-throughput format via fluorogenic thiol quantification (FTQ), in order to determine the kinetics of electrophile-thiol conjugation (Fig. 5) [82]. Control thiols and parallel dynamics analysis can also be used to evaluate the heterogeneous reactivity of covalent electrophilic ligands. Using this method, hit fragments targeting the binding pocket of the protein can be rapidly recognized. Importantly, the site on the protein can be predefined and is not limited by protein function [83]. Thus, the FTQ can be a simple and cost-effective assay for screening covalent inhibitors of PPIs. The above screening strategies can be used to identify covalent inhibitors of PPI targets. The choice of method used will depend on the number of compounds in the library, knowledge of structural information of the inhibitors, and the type of target itself. After screening, covalent inhibition can be confirmed by testing for the time dependency of the IC50 value. If there is no change in the IC50 in the time-dependent assay within a specified time range of the experiment, then it can be inferred that covalent binding between the inhibitor and protein does not take place. This could be, for example, due to the low reactivity of the targeted cysteine residue which hinders covalent bond formation. Looking toward the future, there is still significant room for the development of covalent PPI inhibitors that target non-catalytic, non-cysteine residues. Computational techniques could play an important role in advancing this area by identifying novel hotspots and accelerating the process of inhibitor design.

### Successful covalent PPI inhibitors in cancer therapy

**Fig. 6:**
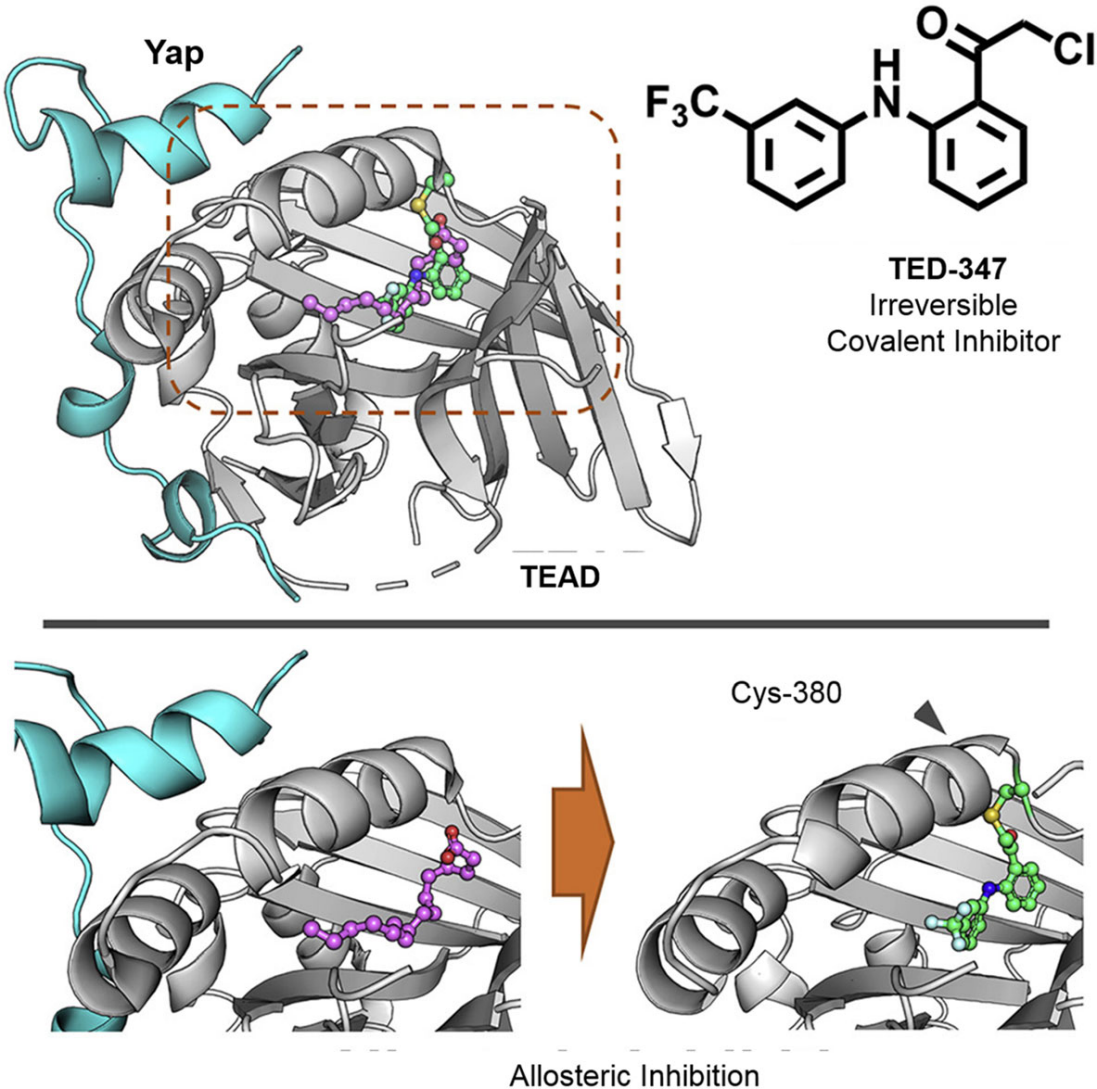
The covalent inhibitor TED-347 inhibits the TEAD-Yap PPI in allosteric formation by targeting a conserved cysteine within the palmitate pocket of TEAD

Although PPIs represent a large class of potential therapeutic targets, the design of effective and selective PPI inhibitors is still challenging. An alternative approach of covalent targeting PPIs has attracted increasing attention as a method of developing more potent PPI inhibitors. In recent years, a number of covalent PPI inhibitors have been approved by the US Food and Drug Administration (FDA) (Table 1). In the specific field of oncology, one covalent PPI inhibitor has been approved by the FDA, while others have entered clinical trials [84, 89, 91–93]. One recent example is the covalent inhibitor COH000, a highly specific allosteric inhibitor of the SUMO (small ubiquitin-related modifier) E1 enzyme, an anticancer target [88]. Interestingly, COH000 binds to a cryptic pocket within SUMO E1 that is separate from its active site and which had been observed to be buried in previous structures of the enzyme. It was hypothesized that the SUMO E1 is in constant structural flux and that COH000 could lock the enzyme into an inactive state that has not been observed before. Most importantly, COH000 has notable anticancer in vitro colorectal cancer cells and in vivo mouse and patient-derived xenograft models [94]. In another work, the covalent inhibitor TED-347 was developed to allosterically target the PPI between transcriptional enhanced associate domains (TEAD) and Yes1- associated protein (Yap), via reacting with a conserved cysteine residue inside the palmitate binding pocket of TEADs (Fig. 6) [90]. In patient-derived glioblastoma spheroids, TED-374-inhibited cell survival could also serve as a cancer probe in the Hippo signaling pathway. The case of covalent PPI inhibitor target TEAD Yap demonstrates that small molecule inhibitors could be more potent for large-scale PPI interfaces with nucleophilic residues and tight pockets on binding site. Myeloid cell leukemia-1 (MCL-1) is one of most frequently amplified genes in cancer due to its role as a survival factor in different kinds of tumors [95, 96]. The first covalent inhibitor targeting Mcl-1 was developed from an aryl boronic acid carbonyl warhead, which reacted with a noncatalytic lysine (Lys234) side chain of the protein. Notably, the inhibitor could activate apoptosis in an Mcl-1-dependent multiple myeloma cell line [47]. Peptide-based covalent PPI inhibitors have also been reported. A reactive peptide derived from an antagonist was designed to target the Grb2−Sos1 PPI through binding to the SH3 domain of Grb2N. The dimeric peptide targeted the nucleophilic Cys32 site of Grb2N-SH3 to form a covalent thioether bond. Moreover, the inhibitor could target endogenous Grb2 and significantly inhibit mobility and viability in SK-BR-3 human breast cancer cells [95]. Although this peptide may not necessarily show features of drug-likeness, it could still be used as an irreversible probe of the Grb2-Sos1 interaction. THZ531, a CDK12-CDK13 covalent inhibitor, binds with CDK12-cyclin K via a cysteine residue that is located outside the kinase domain [97]. THZ531 inhibits hyperphosphorylated and elongating RNA polymerase II, while downregulating DNA damage response genes and associated transcription factor genes [97]. Furthermore, THZ531 induced cell death and apoptosis in Ewing sarcoma cancer cell lines. Developing covalent inhibitors of the CDK12-CDK13 PPI could help identify cancer subtypes that are susceptible to inhibition of this kinase activity [98]. Site-specific strategies can be used to design covalent PPI inhibitors for a wide range of residues. For example, boronic acid carbonyl warheads have been reported to modify the ε-amino group of lysine residues with high binding affinity [99]. This has been used to develop covalent inhibitors of Mcl-1 with biochemical and cellular activity [47]. These warhead covalent inhibitors avoid general cytotoxicity by specifically targeting Mcl-1 to activate caspases. Allosteric inhibitors have also been described that can target “switch II,” an inducible pocket within RAS, but further optimization is required to advance them into clinical trials. Meanwhile, targeted covalent agents have shown potential for treating tumors caused by KRAS G12C [87].

**Table 1:** Recent examples of successfully developed covalent PPI inhibitors

Name	Type	Target	Prototypic cancer		Year	Reference
Stattic	Peptide	HIF-1a/ARNT	Hepatocellular carcinoma		2012	[84]
Aldehyde boronic acid	Small molecule warheads	Mcl-1/Bak	Multiple myeloma		2016	[47]
RP-D	Peptide	Grb2/Sos1	Breast cancer		2017	[85]
Achiral oxoapomorphine	Small molecule inhibitors	P53/MDM2	Prostate cancer		2017	[86]
1_AM	Small molecule inhibitors	KRAS/G12C	Lung cancer		2017	[87]
COH000	Small molecule inhibitors	SUMO E1	Colorectal cancer		2018	[88]
Oridonin	Small molecule inhibitors	NLRP3/NEK7	Breast cancer		2018	[89]
TED-347	Small molecule inhibitors	TEAD/Yap	Glioblastoma		2019	[90]
Aryl-sulfonyl fluorides/aryl-fluoro sulfates	Small molecule warheads	XIAP/BIR3	Ovarian cancer		2019	[61]
cHBIs	Small molecule inhibitors	Allergen/sIgE	Colorectal cancer		2019	[91]

## Conclusion remarks and perspectives

### Perspective and predicament for study PPI inhibitors in cancers

For a long time, PPIs were regarded as “undruggable” targets, despite their importance in the regulation of signaling cascades in myriad diseases, including cancer [13]. With improvements in both chemical and biological technologies, the targeting of PPIs for cancer treatment is becoming an achievable reality. For example, in leukemia, the mixed lineage leukemia (MLL) protein interacts with menin to promote oncogenic activity. Therefore, developing small molecule inhibitors to directly disrupt the MLL-menin interaction is a potential strategy for leukemia treatment [100, 101]. Furthermore, while many cancers are driven by aberrant kinase activity, it is thought that targeting the PPIs that regulate the activity of a specific kinase is a more viable approach to develop selective kinase inhibitors than targeting the conserved ATP binding site of kinases directly [4, 102, 103]. For instance, the heat shock protein (Hsp90)-Cdc37 PPI promotes colorectal cancer. PPI inhibitors of Hsp90-Cdc37 could achieve selective inhibition of Hsp90 kinase without affecting other interactions that could lead to unnecessary toxicity [104]. Another disadvantage of targeting the active site of kinases is that those inhibitors are highly susceptible to resistance arising from mutations in the catalytic domain, such as drug resistance to imatinib in the case of chronic myeloid leukemia patients [4, 105]. However, drawbacks of PPI inhibitors for anticancer therapy are that targeting PPIs has been found to be more difficult than targeting the active sites of enzymes or receptors. As highlighted above, fragment-based screening, computational analysis, and molecular inhibitor design are some of the techniques that have been used in PPI inhibitor development in drug discovery, including in the oncology field [106–110]. Such methods can accelerate the early phases of drug discovery by identifying PPIs as drug targets, followed by physicochemical and topographical characterization of their binding interfaces as well [13, 111, 112]. A number of (mostly non-covalent) PPI inhibitors have already been approved or entered clinical trials for the treatment of cancer (Table 2) [113]. However, non-covalent PPI inhibitors may have the ability to cause “off-target” effects due to binding to other molecules [114–116]. Thus, further research is needed to drive the effect of PPI inhibitors that are both potent and selective for their desired targets.

**Table 2:** Examples of small-molecule development candidates identified against PPIs in cancer treatment

Compound name	Therapeutic area	Highest phase reached
BCL-2 family		
Obatoclax (CEP-41601, GX015-070)	Extensive-stage small cell lung cancer	Phase III (discontinued)
Navitoclax (ABT-263)	Chronic lymphocytic leukemia/prostate cancer	Phase II (completed)
Venetoclax (ABT-199)	Leukemia/acute myeloid leukemia	Phase I
MDM2–p53 pathway		
Idasanutlin (RO5503781)	Leukemia/acute myeloid leukemia	Phase III
AMG232	Metastatic melanoma	Phase I/II
CGM097	Solid tumor with wild-type p53	Phase I
DS-3032b	Advanced solid tumors/lymphomas	Phase I
ALRN-6924	Acute myeloid leukemia /advanced myelodysplastic syndrome	Phase I
MK-8242	Advanced solid tumors	Phase I
JNJ-26854165	Advanced stage or refractory solid tumors	Phase I (completed)
RG-7112 (RO5045337)	Hematologic neoplasms	Phase I (completed)
SAR-405838	Malignant neoplasms	Phase I
IAP pathway		
AT-406 (Debio-1143)	Solid tumor	Phase II
LCL-161	Breast cancer	Phase II
Birinapant (TL32711)	Advanced or metastatic solid tumors	Phase I/II
ASTX-660	Advanced solid tumors and lymphomas	Phase I/II
AEG40826 (HGS1029)	Advanced solid tumors	Phase I
CUDC-427 (GDC-0917)	Lymphoma	Phase I
GDC-0152 (RG-7419)	Advanced or metastatic malignancies	Phase I (discontinued)
GSK525762	Breast cancer	Phase I/II
CPI-0610	Multiple myeloma	Phase I
TEN-010	Acute myeloid leukemia	Phase I
OTX015 (MK-8628)	Hematologic malignancies	Phase I (completed)

#### Perspectives for the future development of covalent PPI inhibitors

**Fig. 7:**
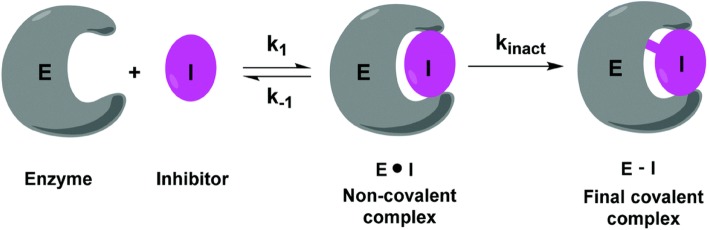
The equations for the target-specific action of covalent inhibitors. E: enzyme target, I: inhibitor, *k*_inact_: rate of inactivation of the target, k2: rate of the subsequent bond-forming reaction, KI: inhibition constant

It is widely recognized that both covalent and non-covalent inhibitors have their respective advantages that can be exploited in different drug modalities, including PPI targeting. For example, non-covalent compound libraries are typically much larger than covalent inhibitors, which may increase the number of leads available for developing PPI inhibition. Non-covalent inhibitors are also thought to be less likely to lead to off-target effects compared to covalent inhibitors. For covalent inhibitors, one of their chief advantages is potentially more durable inhibition at the PPI site leading to improved pharmacology in vivo [117, 118]. With covalent inhibition, the drug action time mainly depends on protein turnover time, rather than other pharmacokinetic properties [119]. This means that covalent PPI inhibitors can be highly potent at relatively low doses. The covalent approach can also be used to target shallow binding sites of receptors, enzymes, and PPI targets that are not conducive for non-covalent targeting. In addition, some analytical techniques such as click chemistry, activity probes, and mass spectrometry-based proteomics to evaluate binding activity and selectivity in vitro and in vivo are only available for covalent compounds [120–124]. The initial binding step (K1) and the subsequent chemical reaction step (*k*_inact_) are two important factors determining the selectivity of covalent inhibitors (Fig. 7) [37]. Having a high Ki is desirable for selectivity and provides sufficient time for the covalent reaction to happen. The rate constant of *k*_inact_ should also be high to drive the reaction to completion during the lifetime of the non-covalent complex. These techniques that have been mentioned above allow for the determining of covalent binding occupancy and biological efficiency in PPIs.

It should be noted that while covalent inhibitors are excellent candidates for disrupting PPIs, they may not be ideal choices for other targets [125, 126]. For example, G-protein-coupled receptors (GPCRs) are influenced by trafficking progress and then internalized in the cell membrane and recycled or degraded. Covalent molecules may not be able to effectively target GPCRs during this turnover process [127]. Moreover, the chief advantage of covalent drugs, i.e., strong binding, may not be desirable for all targets or for all cases. Furthermore, the targets of infectious diseases usually have high replication rates, while covalent inhibitors tend to have long residence times making them less effective in this area [128].

Although there are many potential advantages (e.g., long targeting action and high potency) of covalent PPI inhibitors, their disadvantages should not be neglected. Covalent PPI inhibitors can form chemically reactive metabolites when they bind to the target protein [129], which can lead to severe side effects [130]. For example, the oxidation of drugs such as furosemide, isoniazid, acetaminophen, halothane, and model hepatotoxins via cytochrome P-450 can lead to hepatotoxic effects in animals and humans [131, 132]. Possible toxicity arising from protein haptenization is also a potential risk of covalent PPI inhibitors [133, 134]. In some clinical trials, immunogenicity was observed several weeks or months after treatment with covalent drugs [135, 136]. Additionally, some covalent inhibitors with reactive functional groups tend to be unattractive for drug discovery because of the poor pharmacokinetic values, especially high clearance [137]. Thus, it is necessary to develop strategies to evaluate the likelihood of covalent inhibitors undergoing metabolism in order to prevent drug toxicity as much as possible at the early stage of covalent PPI inhibitor development [138]. Several approaches have recently been reported in order to minimize the risk of side effects of covalent PPI agents. For instance, some covalent bifunctional blockers are designed with deliberately weak reactivity in order to increase stability and to decrease toxicity. In some cases, covalent inhibitors can be designed to be cleared rapidly after blocking the PPI, which could reduce non-mechanism-based toxicity [37, 139]. Overcoming toxicity is one of the main bottlenecks preventing covalent inhibitors from entering the market [140]. A summary of the advantages and disadvantages of covalent and non-covalent PPI inhibitors is presented in Table 3.

**Table 3:** The advantages and disadvantages of covalent and noncovalent PPI inhibitors

	Advantages	Disadvantages
Covalent PPI inhibitors	Longer duration of action Lower prolonged systemic exposure Higher potency and selectivity Higher biochemical efficiency Less sensitive to pharmacokinetic parameters Computational prediction of reversible covalent binding Lower risk of drug resistance	Off-target toxicity Potential immunogenicity of the resulting target adducts
Non-covalent PPI inhibitors	Larger non-covalent compound libraries Long dissociated half-lives Less off-target toxicity	Not very selective Usually weak reactivity Limit to non-covalent binding affinity Not very potent

Additionally, precise mechanisms by which covalent inhibitors form covalent bonds with their target proteins to exert pharmacological and toxicological effects are still unclear. Therefore, shedding light on the potential mechanisms of pharmacology and toxicology of covalent PPI inhibitors is an important directive to facilitate drug development. Moreover, frameworks to evaluate the reactivity profiles, efficiency, selectivity, and toxicity of covalent inhibitors need to be established. Overcoming these challenges will provide renewed impetus for scientists to explore novel covalent inhibitors targeting PPI for various human diseases. In conclusion, we believe that covalent PPI inhibitors have a promising future and more of such therapeutic agents will be developed in the coming years.

## Data Availability

Not applicable.

## Abbreviations

ABPP: Activity-based protein profiling
ABPs: Activity-based probes
AVPF: Ala-Val-Pro-Phe
FDA: Food and Drug Administration
FTQ: Fluorogenic thiol quantification
GPCRs: G-protein-coupled receptors
Hsp90: Heat shock protein 90
HTS: High-throughput screening
isoTOP-ABPP: Isotopic tandem orthogonal proteolysis activity-based protein profiling
MCL-1: Myeloid cell leukemia-1
MD: Molecular dynamics
PPIs: Protein-protein interactions
qIT: Quantitative irreversible tethering
QSAR: Quantitative structure activity relationships
SMAC: Second mitochondria-derived activator of caspase
SUMO: Small ubiquitin-related modifier
TEAD: Transcriptional enhanced associate domains
XIAP: X-linked inhibitor of apoptosis protein
Yap: Yes1-associated protein
