# Correction to: The design and development of covalent protein-protein interaction inhibitors for cancer treatment

**DOI:** 10.1186/s13045-020-00938-7

**Published:** 2020-07-24

**Authors:** Sha-Sha Cheng, Guan-Jun Yang, Wanhe Wang, Chung-Hang Leung, Dik-Lung Ma

**Affiliations:** 1 State Key Laboratory of Quality Research in Chinese Medicine, Institute of Chinese Medical Sciences, University of Macau, Macao, 999078 China; 2 Department of Chemistry, Hong Kong Baptist University, Hong Kong, 999077 China

**Correction to: J Hematol Oncol 13, 26 (2020)**

**https://doi.org/10.1186/s13045-020-00850-0**

The original article [1] contains an omission in the legend of Fig. 2 whereby inadequate credit was given to the authors of the original print of Fig. 2 which was reproduced from the original article published in *Medicinal Research Reviews* [2].

**Fig. 2:**
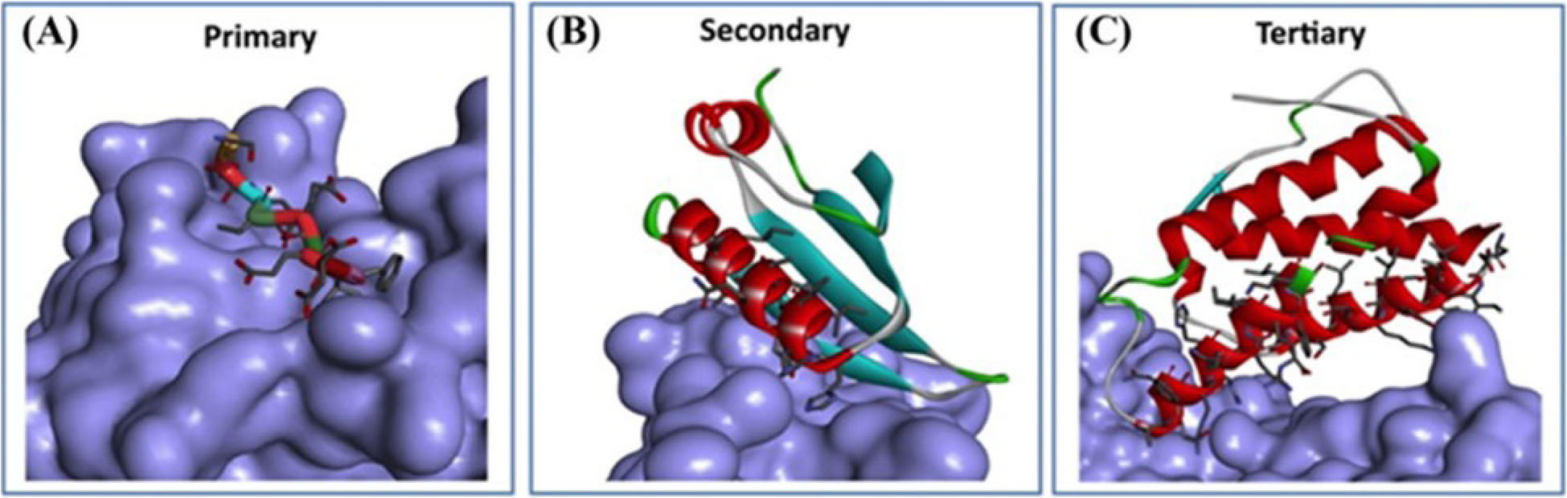
The crystal structure of three different classes of PPIs [2]. A) Linear sequences comprise primary peptide epitopes (e.g. LM of DNA polymerase III bound to the binding pocket of the SC (PDB: 3D1F)); B) the secondary structure of epitopes binds as a single unit. e.g. an α-helix (NusB-NusE PPI (PDB: 3R2C)); and C) in tertiary structural epitopes, the binding interface is not continuous and both sides of PPI interface are needed (e.g. IL-2/IL-2Ra PPI (PDB 1Z92))

The correct version of Fig. 2’s legend with appropriate citation can be viewed ahead alongside its respective figure.
